# Crystal structure of (*R*)-6′-bromo-3,3-dimethyl-3′,4′-di­hydro-2′*H*-spiro­[cyclo­hexane-1,3′-1,2,4-benzo­thia­diazine] 1′,1′-dioxide

**DOI:** 10.1107/S1600536814022417

**Published:** 2014-10-18

**Authors:** P. P. Shinoj Kumar, P. A. Suchetan, S. Sreenivasa, S. Naveen, N. K. Lokanath, D. B. Aruna Kumar

**Affiliations:** a Department of Studies and Research in Chemistry, Tumkur University, Tumkur 572 103, India; b Department of Studies and Research in Chemistry, U.C.S., Tumkur University, Tumkur 572 013, India; c Institution of Excellence, Vijnana Bhavan, University of Mysore, Manasagangotri, India; d Department of Studies in Physics, University of Mysore, Manasagangotri, Mysore, India

**Keywords:** crystal structure, benzo­thia­diazine, hydrogen bonding, chirality

## Abstract

In the title compound, C_14_H_19_BrN_2_O_2_S, the 1,2,4-thia­diazinane ring adopts an envelope conformation with the N atom (attached to the sulfonyl group) as the flap, while the cyclo­hexane ring adopts a chair conformation. The mean plane of the cyclo­hexane ring is almost normal to the benzene ring and the mean plane of the 1,2,4-thia­diazinane ring, making dihedral angles of 70.4 (2) and 71.43 (19)°, respectively. Furthermore, the dihedral angle between the benzene ring and the mean plane of the 1,2,4-thia­diazinane ring is 4.91 (18)°. The mol­ecular structure is stabilized by an intra­molecular C—H⋯O hydrogen bond, which encloses an *S*(6) ring motif. In the crystal, mol­ecules are linked by N—H⋯O hydrogen bonds into chains along [10-1], forming a *C*(6) graph-set motif. These chains are inter­connected *via* C—H⋯π inter­actions, leading to chains along [-101], so finally forming sheets parallel to (010).

## Abstract 2

In the title compound, the mean plane of the cyclo­hexane ring is almost normal to the benzene ring and to the mean plane of the 1,2,4-thia­diazinane ring. In the crystal, mol­ecules are linked by N—H⋯O hydrogen bonds, forming chains along [10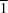], which are in turn linked *via* C—H⋯π inter­actions, forming sheets parallel to (010).

## Chemical context   

The sulfonamide class of drugs have been widely reported for their anti­bacterial and anti­fungal activities (Trujillo *et al.*, 2009 ▶). 1,2,4-Benzo­thia­diazine 1,1-dioxides are used as anti­hypertensive, diuretic, anti­diabetic, glutamine­rgic neuro modulators (Cordi *et al.*, 1996 ▶) and K-channel inhibitors (Di Bella *et al.*, 1983 ▶). Furthermore, benzo­thia­diazine-3-one 1,1-dioxide and its derivatives have been reported for their potential hypoglycemic (Scozzafava *et al.*, 2003 ▶), anti­cancer and anti-HIV activities (Casini *et al.*, 2002 ▶), and they have also been reported to serve as selective antagonists of CXR2 (Hayao *et al.*, 1968 ▶). In addition, 2-substituted-2*H*-1,2,4-benzo­thia­diazine-3(4*H*)one 1,1-dioxides have been found to exhibit varying degrees of sedative and hypotensive activities (Khelili *et al.*, 2012 ▶). A number of benzo­thia­diazine 1,1-dioxide derivatives have recently been reported to display numerous biological activities (Tullio *et al.*, 2011 ▶). 
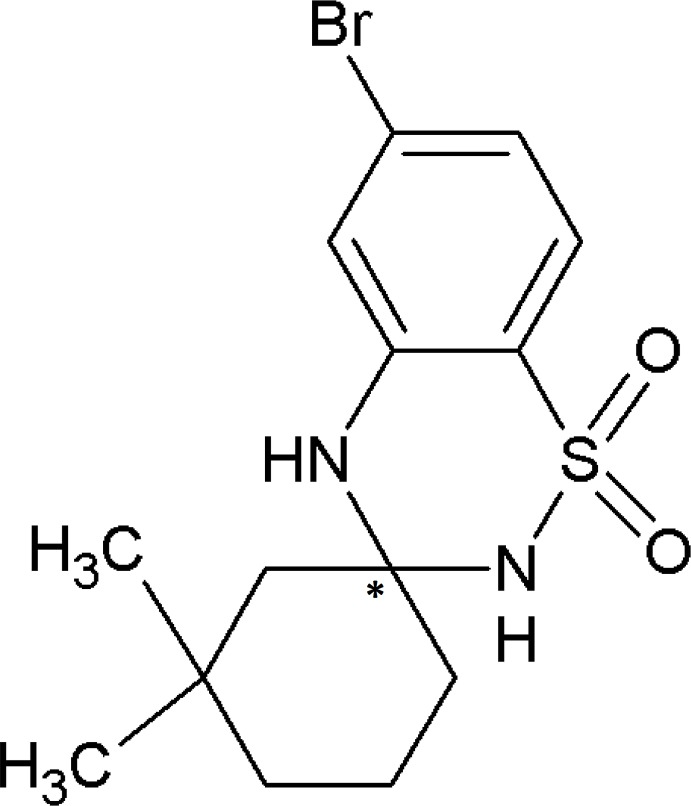


In view of their broad spectrum of biological activities, and in a continuation of our work on this class of compound, we have synthesized the title compound, (1), and report herein on its spectroscopic analysis and crystal structure.

## Structural commentary   

**Figure 1:**
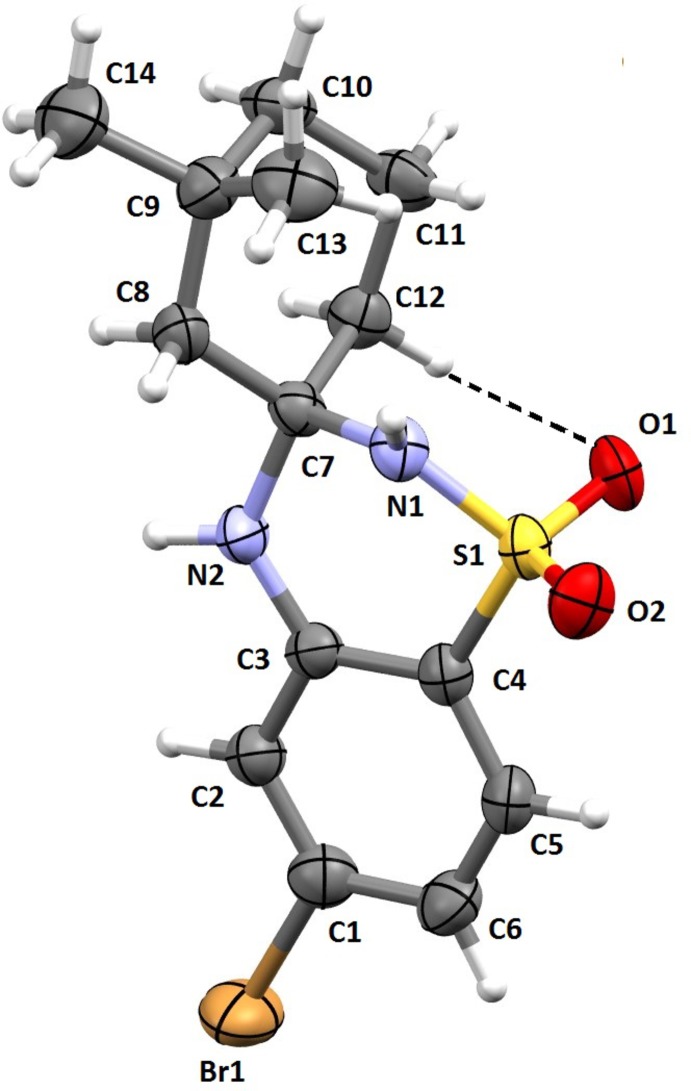
A view of the mol­ecular structure of the title mol­ecule, showing the atom labelling. Displacement ellipsoids are drawn at the 50% probability level. The C—H⋯O hydrogen bond is shown as a dashed line (see Table 1 ▶ for details).

The mol­ecular structure of the title mol­ecule is shown in Fig. 1 ▶. The relative configuration of the asymmetric center is *R* for atom C7. The cyclo­hexane ring (C7–C12) adopts a chair conformation, confirmed by the puckering amplitude of *Q* = 0.4285 Å. The 1,2,4-thia­diazinane ring (N1/S1/C4/C3/N2/C7) adopts an envelope conformation with the flap atom N1 deviating by 0.565 (3) Å from the mean plane defined by atoms C7/N2/C3/C4/S1 [maximum deviation = 0.033 (1) Å for atom S1]. The mean plane of the cyclo­hexane ring is almost normal to the benzene ring (C1–C6) and the mean plane of the 1,2,4-thia­diazinane ring, making dihedral angles of 70.4 (2) and 71.43 (19)°, respectively. The dihedral angle between the benzene ring and the mean plane of the 1,2,4-thia­diazinane ring is 4.91 (18)°. The mol­ecular structure is stabilized by an intra­molecular C—H⋯O hydrogen bond, which forms an *S*(6) ring motif (Table 1 ▶ and Fig. 1 ▶).

## Supra­molecular features   

**Figure 2:**
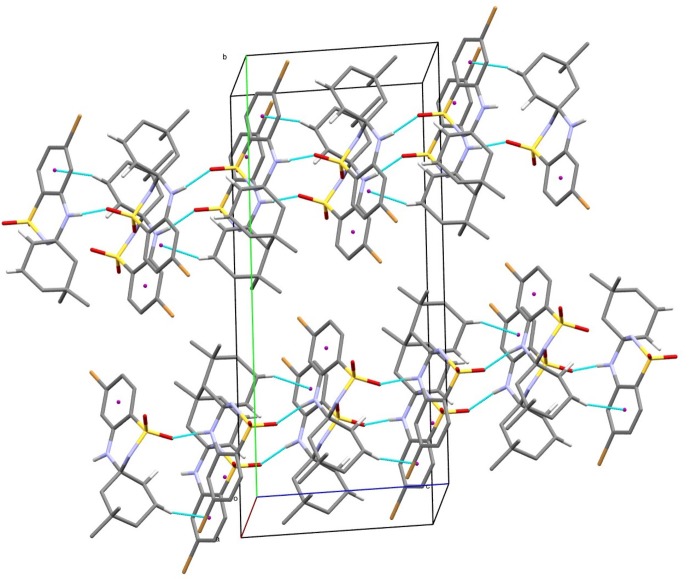
A view along the *a* axis of the crystal packing of the title compound. Hydrogen bonds are shown as thin blue lines (see Table 1 ▶ for details; H atoms not involved in hydrogen bonding have been omitted for clarity).

In the crystal, mol­ecules are linked by N—H⋯O hydrogen bonds (Table 1 ▶ and Fig. 2 ▶), forming chains with a *C*(6) graph-set motif along [10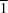]. The chains are linked *via* structure-directing C—H⋯π inter­actions, leading to the formation of *C*(6) chains along [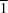
01]. These inter­actions lead to the formation of sheets parallel to (010) (Table 1 ▶ and Fig. 2 ▶).

## Database survey   

In two similar structures, namely 6-bromo-4*H*-spiro­[1,2,4-benzo­thia­diazine-3,1′-cyclo­butane] 1,1-dioxide, (2) (Shinoj Kumar, 2014*a* ▶, and 6-bromo-1′-ethyl-4*H*-spiro­[1,2,4-benzo­thia­diazine-3,4′-piperidine] 1,1-dioxide, (3) (Shinoj Kumar, 2014*b* ▶, the 1,2,4-thia­diazinane rings adopt a twisted chair conformation, in contrast to the envelope conformation observed in (1). In (2), the dihedral angle between the benzene ring and the mean plane of the cyclo­butyl ring is 73.76 (1)°, while that between the benzene ring and the mean plane of the 1,2,4-thia­diazinane ring is 4.72 (1)°, and that between the mean plane of the cyclo­butyl ring and the mean plane of the 1,2,4-thia­diazinane ring is 78.44 (1)°. In (3), the same dihedral angles are similar, being 73.61 (1), 6.73 (1) and 73.81 (1)°, respectively. These angles are also similar to those observed in the title compound, (1).

## Synthesis and crystallization   

To a cooled solution of 2-amino-4-bromo­benzene sulfonamide (5 g, 19.9 mmol) and anhydrous magnesium sulfate (MgSO_4_; 3.5 g, 29.88 mmol) in dry toluene (60 ml), 3,3-di­methyl­cyclo­hexa­none (22 mmol) was added followed by slow addition of polyphospho­ric acid anhydride (T3P; 19 ml, 29.88 mmol, 50% solution in ethyl acetate). The reaction mixture was then refluxed in a sealed tube at 393 K for 6 h. It was cooled to 283 K and neutralized with saturated sodium bicarbonate solution (100 ml). The crude product was extracted with ethyl acetate (100 ml) and was finally washed with brine solution (50 ml). The organic phase was dried over anhydrous sodium sulfate and concentrated to give the crude product as a brown solid. It was then dissolved in a minimum amount of ethyl acetate (25 ml) and stirred for 1h in an ice-cooled bath, filtered and washed with cold ethyl acetate (10 ml × 2) to give pure compound (1) (4.5 g, 75% yield) as a white solid. Colourless prisms of the title compound were obtained by slow evaporation of a solution of the compound in ethyl acetate.

## Spectroscopic characterization   

The IR spectra of the title compound exhibits strong bands at 1374 cm^−1^ due to asymmetric (S=O) stretching and a band at 1165 cm^−1^ due to symmetric (S=O) stretching. Further, a single band appearing at 3110 cm^−1^ is due to the secondary N—H group of the sulfonamide, and a band at 3308 cm^−1^ confirms the cyclization of sulfonamide through condensation with the ketone. Appearance of bands in the range of 2970–2815 cm^−1^ is assigned to the C—H stretching is due to the presence of the saturated hydro­carbons. The ^1^H NMR spectrum shows peaks at δ 7.53 (*s*, 1H, SO_2_NH), 6.934–6.930 (*d*, 1H, Ar-H), 7.37–7.35 (*d*, 1H, Ar-H), 3.33 (*s*, 1H, NH), 2.51–1.28 (*m*, 9H, CH_2_), 0.9–1.1 (*s*, 6H, CH_3_). The ^13^C NMR spectrum shows peaks at δ 144 (C1), 119 (C2), 126 (C3), 127 (C4), 119 (C5), 118 (C6), 117 (C7), 71 (C8), 47 (C9), 36 (C10), 33 (C11), 31 (C12), 18 (C13 and C14). The LC–MS spectrum shows the appearance of mol­ecular ion peaks at *m*/*z* 358 and 357 values, confirming the structure of the compound.

## Refinement   

Crystal data, data collection and structure refinement details are summarized in Table 2 ▶. The NH hydrogens were located in a difference Fourier map. N- and C-bound H atoms were included in calculated positions (N—H = 0.86 and C—H = 0.93–0.97 Å) and allowed to ride on their parent atoms, with *U*_iso_(H) = 1.5*U*_eq_(C) for methyl H atoms and 1.2*U*_eq_(N,C) for other H atoms.

## Supplementary Material

Crystal structure: contains datablock(s) 1, Global. DOI: 10.1107/S1600536814022417/su2797sup1.cif

Structure factors: contains datablock(s) 1. DOI: 10.1107/S1600536814022417/su27971sup2.hkl

CCDC reference: 1028895

Additional supporting information:  crystallographic information; 3D view; checkCIF report

## Figures and Tables

**Table 1:** Hydrogen-bond geometry (, ) *Cg* is the centroid of the C1C6 ring.

*D*H*A*	*D*H	H*A*	*D* *A*	*D*H*A*
C12H12*A*O1	0.97	2.40	3.066(5)	126
N2H*N*2O1^i^	0.86	2.26	3.101(5)	166
C11H11*A* *Cg* ^ii^	0.97	2.58	3.506(5)	160

Symmetry codes: (i) 
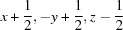
; (ii) 
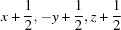
.

**Table 2:** Experimental details

Crystal data	
Chemical formula	C_14_H_19_BrN_2_O_2_S
*M*_r_	359.28
Crystal system, space group	Monoclinic, *P*2_1_/*n*
Temperature (K)	293
*a*, *b*, *c* ()	6.4316(7), 24.263(3), 10.0829(13)
()	105.604(9)
*V* (^3^)	1515.5(3)
*Z*	4
Radiation type	Cu *K*
(mm^1^)	5.01
Crystal size (mm)	0.44 0.24 0.19

Data collection	
Diffractometer	Bruker APEXII
Absorption correction	Multi-scan (*SADABS*; Bruker, 2009 ▶)
*T*_min_, *T*_max_	0.271, 0.386
No. of measured, independent and observed [*I* > 2(*I*)] reflections	11574, 2515, 1860
*R*_int_	0.081
(sin /)_max_ (^1^)	0.586

Refinement	
*R*[*F* ^2^ > 2(*F* ^2^)], *wR*(*F* ^2^), *S*	0.049, 0.154, 0.94
No. of reflections	2515
No. of parameters	183
H-atom treatment	H-atom parameters constrained
_max_, _min_ (e ^3^)	0.61, 0.61

Computer programs: *APEX2*, *SAINT-Plus* and *XPREP* (Bruker, 2009 ▶), *SHELXS97* and *SHELXL97* (Sheldrick, 2008 ▶), and*Mercury* (Macrae *et al.*, 2008 ▶).

## supplementary crystallographic information

### Crystal data

**Table:**

C_14_H_19_BrN_2_O_2_S	*D*_x_ = 1.575 Mg m^−^^3^
*M**_r_* = 359.28	Melting point: 418 K
Monoclinic, *P*2_1_/*n*	Cu *K*α radiation, λ = 1.54178 Å
*a* = 6.4316 (7) Å	Cell parameters from 123 reflections
*b* = 24.263 (3) Å	θ = 7.1–64.6°
*c* = 10.0829 (13) Å	µ = 5.01 mm^−^^1^
β = 105.604 (9)°	*T* = 293 K
*V* = 1515.5 (3) Å^3^	Prism, colourless
*Z* = 4	0.44 × 0.24 × 0.19 mm
*F*(000) = 736	

### Data collection

**Table:**

Bruker APEXII diffractometer	1860 reflections with *I* > 2σ(*I*)
Radiation source: fine-focus sealed tube	*R*_int_ = 0.081
Graphite monochromator	θ_max_ = 64.6°, θ_min_ = 7.1°
phi and φ scans	*h* = −7→6
Absorption correction: multi-scan (*SADABS*; Bruker, 2009)	*k* = −28→27
*T*_min_ = 0.271, *T*_max_ = 0.386	*l* = −11→11
11574 measured reflections	1 standard reflections every 1 reflections
2515 independent reflections	intensity decay: 1%

### Refinement

**Table:**

Refinement on *F*^2^	Primary atom site location: structure-invariant direct methods
Least-squares matrix: full	Secondary atom site location: difference Fourier map
*R*[*F*^2^ > 2σ(*F*^2^)] = 0.049	Hydrogen site location: inferred from neighbouring sites
*wR*(*F*^2^) = 0.154	H-atom parameters constrained
*S* = 0.94	*w* = 1/[σ^2^(*F*_o_^2^) + (0.1058*P*)^2^ + 0.1836*P*] where *P* = (*F*_o_^2^ + 2*F*_c_^2^)/3
2515 reflections	(Δ/σ)_max_ < 0.001
183 parameters	Δρ_max_ = 0.61 e Å^−^^3^
0 restraints	Δρ_min_ = −0.61 e Å^−^^3^

### Special details

**Table:**

Experimental. Melting points were determined in open capillaries and are uncorrected. The molecular structures of the synthesized compounds were established using IR, ^1^H NMR, ^13^C NMR and LC-MS studies. Solid state FT-IR Spectra were recorded as KBr discs on Jasco FT-IR Spectrometer. ^1^H NMR and ^13^C NMR were recorded in DMSO at 399.13 MHz and 75.50 MHz respectively on Bruker model avance II. All the chemical shifts were reported in parts per million (ppm) using tetramethyl silane (TMS) as an internal standard. Mass spectra of the compounds were recordedon Shimadzu LC-2010EV with ESI probe.
Geometry. All s.u.'s (except the s.u. in the dihedral angle between two l.s. planes) are estimated using the full covariance matrix. The cell s.u.'s are taken into account individually in the estimation of s.u.'s in distances, angles and torsion angles; correlations between s.u.'s in cell parameters are only used when they are defined by crystal symmetry. An approximate (isotropic) treatment of cell s.u.'s is used for estimating s.u.'s involving l.s. planes.
Refinement. Refinement of *F*^2^ against ALL reflections. The weighted *R*-factor *wR* and goodness of fit *S* are based on *F*^2^, conventional *R*-factors *R* are based on *F*, with *F* set to zero for negative *F*^2^. The threshold expression of *F*^2^ > 2σ(*F*^2^) is used only for calculating *R*-factors(gt) *etc*. and is not relevant to the choice of reflections for refinement. *R*-factors based on *F*^2^ are statistically about twice as large as those based on *F*, and *R*- factors based on ALL data will be even larger.

### Fractional atomic coordinates and isotropic or equivalent isotropic displacement parameters (Å^2^)

**Table:**

	*x*	*y*	*z*	*U*_iso_*/*U*_eq_	
C1	0.0489 (7)	0.09465 (19)	0.8681 (5)	0.0449 (11)	
C2	0.0873 (7)	0.14863 (18)	0.8441 (4)	0.0398 (10)	
H2	0.1873	0.1575	0.7959	0.048*	
C3	−0.0244 (6)	0.19093 (16)	0.8922 (4)	0.0336 (9)	
C4	−0.1634 (6)	0.17472 (17)	0.9715 (4)	0.0334 (9)	
C5	−0.2014 (7)	0.11957 (18)	0.9908 (4)	0.0419 (10)	
H5	−0.2992	0.1101	1.0401	0.050*	
C6	−0.0993 (8)	0.07862 (19)	0.9394 (5)	0.0465 (11)	
H6	−0.1275	0.0416	0.9514	0.056*	
C7	−0.0697 (6)	0.29279 (16)	0.9203 (4)	0.0327 (9)	
C8	−0.1071 (7)	0.33969 (17)	0.8148 (4)	0.0392 (10)	
H8A	−0.2288	0.3297	0.7384	0.047*	
H8B	0.0186	0.3419	0.7792	0.047*	
C9	−0.1502 (7)	0.39760 (18)	0.8637 (5)	0.0444 (11)	
C10	0.0144 (8)	0.41054 (18)	1.0010 (5)	0.0491 (11)	
H10A	−0.0274	0.4444	1.0382	0.059*	
H10B	0.1547	0.4165	0.9848	0.059*	
C11	0.0326 (8)	0.36432 (19)	1.1071 (4)	0.0464 (11)	
H11A	0.1388	0.3744	1.1918	0.056*	
H11B	−0.1053	0.3595	1.1277	0.056*	
C12	0.0987 (6)	0.31045 (18)	1.0527 (4)	0.0378 (10)	
H12A	0.1127	0.2819	1.1220	0.045*	
H12B	0.2379	0.3150	1.0339	0.045*	
C13	−0.3793 (8)	0.4038 (2)	0.8792 (6)	0.0579 (13)	
H13A	−0.3914	0.3838	0.9590	0.087*	
H13B	−0.4809	0.3893	0.7988	0.087*	
H13C	−0.4094	0.4420	0.8895	0.087*	
C14	−0.1192 (10)	0.4391 (2)	0.7572 (6)	0.0657 (15)	
H14A	−0.1452	0.4756	0.7856	0.098*	
H14B	−0.2187	0.4311	0.6697	0.098*	
H14C	0.0259	0.4366	0.7492	0.098*	
N1	−0.2810 (5)	0.27879 (14)	0.9457 (3)	0.0342 (8)	
HN1	−0.3930	0.2988	0.9117	0.041*	
N2	0.0041 (5)	0.24419 (14)	0.8593 (4)	0.0373 (8)	
HN2	0.0712	0.2499	0.7974	0.045*	
O1	−0.1804 (5)	0.23557 (14)	1.1797 (3)	0.0442 (8)	
O2	−0.5208 (4)	0.21181 (13)	1.0131 (3)	0.0469 (8)	
S1	−0.29684 (15)	0.22511 (4)	1.03879 (10)	0.0352 (3)	
Br1	0.20573 (10)	0.03945 (2)	0.80348 (7)	0.0701 (3)	

### Atomic displacement parameters (Å^2^)

**Table:**

	*U*^11^	*U*^22^	*U*^33^	*U*^12^	*U*^13^	*U*^23^
C1	0.043 (3)	0.043 (3)	0.047 (3)	−0.001 (2)	0.010 (2)	−0.002 (2)
C2	0.041 (2)	0.038 (2)	0.044 (2)	−0.0026 (19)	0.0185 (19)	−0.0035 (18)
C3	0.035 (2)	0.031 (2)	0.033 (2)	−0.0009 (17)	0.0070 (16)	0.0000 (16)
C4	0.032 (2)	0.040 (2)	0.030 (2)	−0.0002 (17)	0.0097 (16)	0.0006 (16)
C5	0.048 (3)	0.045 (2)	0.036 (2)	−0.002 (2)	0.0182 (19)	0.0046 (19)
C6	0.057 (3)	0.037 (2)	0.046 (3)	−0.001 (2)	0.013 (2)	0.008 (2)
C7	0.030 (2)	0.034 (2)	0.035 (2)	0.0003 (17)	0.0117 (16)	−0.0038 (16)
C8	0.046 (2)	0.041 (3)	0.032 (2)	−0.006 (2)	0.0119 (18)	−0.0009 (18)
C9	0.052 (3)	0.037 (2)	0.044 (3)	−0.003 (2)	0.013 (2)	0.0022 (19)
C10	0.058 (3)	0.037 (3)	0.049 (3)	−0.004 (2)	0.010 (2)	−0.010 (2)
C11	0.050 (3)	0.048 (3)	0.034 (2)	−0.002 (2)	0.0005 (19)	−0.009 (2)
C12	0.030 (2)	0.043 (2)	0.037 (2)	−0.0027 (18)	0.0044 (17)	0.0009 (18)
C13	0.049 (3)	0.055 (3)	0.067 (3)	0.014 (2)	0.009 (2)	0.001 (3)
C14	0.091 (4)	0.052 (3)	0.051 (3)	0.000 (3)	0.013 (3)	0.011 (2)
N1	0.0267 (16)	0.040 (2)	0.0370 (19)	0.0074 (14)	0.0098 (14)	0.0041 (15)
N2	0.0420 (19)	0.0343 (19)	0.044 (2)	−0.0006 (15)	0.0265 (16)	−0.0026 (15)
O1	0.0478 (18)	0.060 (2)	0.0258 (15)	0.0024 (15)	0.0112 (13)	−0.0019 (13)
O2	0.0286 (15)	0.058 (2)	0.0562 (19)	−0.0040 (14)	0.0153 (13)	0.0033 (15)
S1	0.0322 (5)	0.0435 (6)	0.0322 (6)	0.0004 (4)	0.0126 (4)	0.0007 (4)
Br1	0.0868 (5)	0.0414 (4)	0.0966 (6)	0.0091 (3)	0.0495 (4)	−0.0070 (3)

### Geometric parameters (Å, º)

**Table:**

C1—C2	1.366 (6)	C9—C10	1.533 (6)
C1—C6	1.394 (6)	C10—C11	1.533 (6)
C1—Br1	1.894 (5)	C10—H10A	0.9700
C2—C3	1.412 (6)	C10—H10B	0.9700
C2—H2	0.9300	C11—C12	1.522 (6)
C3—N2	1.359 (5)	C11—H11A	0.9700
C3—C4	1.407 (5)	C11—H11B	0.9700
C4—C5	1.383 (6)	C12—H12A	0.9700
C4—S1	1.733 (4)	C12—H12B	0.9700
C5—C6	1.368 (6)	C13—H13A	0.9600
C5—H5	0.9300	C13—H13B	0.9600
C6—H6	0.9300	C13—H13C	0.9600
C7—N2	1.466 (5)	C14—H14A	0.9600
C7—N1	1.489 (5)	C14—H14B	0.9600
C7—C8	1.532 (6)	C14—H14C	0.9600
C7—C12	1.537 (5)	N1—S1	1.624 (3)
C8—C9	1.539 (6)	N1—HN1	0.8600
C8—H8A	0.9700	N2—HN2	0.8600
C8—H8B	0.9700	O1—S1	1.439 (3)
C9—C13	1.530 (6)	O2—S1	1.430 (3)
C9—C14	1.523 (6)		
			
C2—C1—C6	122.7 (4)	C9—C10—H10B	109.1
C2—C1—Br1	118.6 (3)	C11—C10—H10B	109.1
C6—C1—Br1	118.8 (4)	H10A—C10—H10B	107.8
C1—C2—C3	120.2 (4)	C12—C11—C10	110.7 (4)
C1—C2—H2	119.9	C12—C11—H11A	109.5
C3—C2—H2	119.9	C10—C11—H11A	109.5
N2—C3—C2	119.5 (4)	C12—C11—H11B	109.5
N2—C3—C4	123.6 (4)	C10—C11—H11B	109.5
C2—C3—C4	116.9 (4)	H11A—C11—H11B	108.1
C5—C4—C3	120.9 (4)	C11—C12—C7	110.7 (3)
C5—C4—S1	120.2 (3)	C11—C12—H12A	109.5
C3—C4—S1	118.8 (3)	C7—C12—H12A	109.5
C6—C5—C4	121.9 (4)	C11—C12—H12B	109.5
C6—C5—H5	119.0	C7—C12—H12B	109.5
C4—C5—H5	119.0	H12A—C12—H12B	108.1
C5—C6—C1	117.2 (4)	C9—C13—H13A	109.5
C5—C6—H6	121.4	C9—C13—H13B	109.5
C1—C6—H6	121.4	H13A—C13—H13B	109.5
N2—C7—N1	107.6 (3)	C9—C13—H13C	109.5
N2—C7—C8	108.3 (3)	H13A—C13—H13C	109.5
N1—C7—C8	108.0 (3)	H13B—C13—H13C	109.5
N2—C7—C12	110.9 (3)	C9—C14—H14A	109.5
N1—C7—C12	112.1 (3)	C9—C14—H14B	109.5
C8—C7—C12	109.8 (3)	H14A—C14—H14B	109.5
C7—C8—C9	117.6 (3)	C9—C14—H14C	109.5
C7—C8—H8A	107.9	H14A—C14—H14C	109.5
C9—C8—H8A	107.9	H14B—C14—H14C	109.5
C7—C8—H8B	107.9	C7—N1—S1	119.0 (3)
C9—C8—H8B	107.9	C7—N1—HN1	120.5
H8A—C8—H8B	107.2	S1—N1—HN1	120.5
C13—C9—C14	108.7 (4)	C3—N2—C7	125.6 (3)
C13—C9—C10	109.8 (4)	C3—N2—HN2	117.2
C14—C9—C10	108.2 (4)	C7—N2—HN2	117.2
C13—C9—C8	112.6 (4)	O2—S1—O1	116.72 (18)
C14—C9—C8	107.9 (4)	O2—S1—N1	107.04 (18)
C10—C9—C8	109.6 (4)	O1—S1—N1	109.40 (19)
C9—C10—C11	112.7 (4)	O2—S1—C4	110.51 (19)
C9—C10—H10A	109.1	O1—S1—C4	109.26 (18)
C11—C10—H10A	109.1	N1—S1—C4	103.01 (17)
			
C6—C1—C2—C3	0.2 (7)	C9—C10—C11—C12	58.9 (5)
Br1—C1—C2—C3	−179.2 (3)	C10—C11—C12—C7	−60.5 (5)
C1—C2—C3—N2	−175.4 (4)	N2—C7—C12—C11	173.9 (3)
C1—C2—C3—C4	3.8 (6)	N1—C7—C12—C11	−65.8 (4)
N2—C3—C4—C5	173.8 (4)	C8—C7—C12—C11	54.3 (4)
C2—C3—C4—C5	−5.3 (6)	N2—C7—N1—S1	55.6 (4)
N2—C3—C4—S1	−3.3 (5)	C8—C7—N1—S1	172.3 (3)
C2—C3—C4—S1	177.6 (3)	C12—C7—N1—S1	−66.6 (4)
C3—C4—C5—C6	2.9 (6)	C2—C3—N2—C7	−168.8 (4)
S1—C4—C5—C6	−180.0 (3)	C4—C3—N2—C7	12.1 (6)
C4—C5—C6—C1	1.1 (7)	N1—C7—N2—C3	−36.3 (5)
C2—C1—C6—C5	−2.7 (7)	C8—C7—N2—C3	−152.8 (4)
Br1—C1—C6—C5	176.7 (3)	C12—C7—N2—C3	86.6 (5)
N2—C7—C8—C9	−170.4 (3)	C7—N1—S1—O2	−163.1 (3)
N1—C7—C8—C9	73.3 (4)	C7—N1—S1—O1	69.6 (3)
C12—C7—C8—C9	−49.2 (5)	C7—N1—S1—C4	−46.5 (3)
C7—C8—C9—C13	−75.8 (5)	C5—C4—S1—O2	−44.6 (4)
C7—C8—C9—C14	164.3 (4)	C3—C4—S1—O2	132.5 (3)
C7—C8—C9—C10	46.7 (5)	C5—C4—S1—O1	85.1 (4)
C13—C9—C10—C11	74.3 (5)	C3—C4—S1—O1	−97.7 (3)
C14—C9—C10—C11	−167.3 (4)	C5—C4—S1—N1	−158.6 (3)
C8—C9—C10—C11	−49.9 (5)	C3—C4—S1—N1	18.5 (3)

### Hydrogen-bond geometry (Å, º)

Cg is the centroid of the C1–C6 ring.

**Table:**

*D*—H···*A*	*D*—H	H···*A*	*D*···*A*	*D*—H···*A*
C12—H12*A*···O1	0.97	2.40	3.066 (5)	126
N2—H*N*2···O1^i^	0.86	2.26	3.101 (5)	166
C11—H11*A*···*Cg*^ii^	0.97	2.58	3.506 (5)	160

Symmetry codes: (i) *x*+1/2, −*y*+1/2, *z*−1/2; (ii) *x*+1/2, −*y*+1/2, *z*+1/2.
